# Impact of pre-treatment extracellular volume fraction measured by computed tomography on response of primary lesion to preoperative chemotherapy in abdominal neuroblastoma

**DOI:** 10.1016/j.clinsp.2024.100434

**Published:** 2024-07-02

**Authors:** Haoru Wang, Xin Chen, Mingye Xie, Jinjie Qin, Ting Li, Ling He

**Affiliations:** Department of Radiology, Children's Hospital of Chongqing Medical University, National Clinical Research Center for Child Health and Disorders, Ministry of Education Key Laboratory of Child Development and Disorders, Chongqing Key Laboratory of Child Neurodevelopment and Cognitive Disorders, Yuzhong District, China

**Keywords:** Neuroblastoma, Extracellular Volume Fraction, Preoperative Chemotherapy, Computed Tomography

## Abstract

**Objectives:**

To retrospectively investigate the impact of pre-treatment Extracellular Volume Fraction (ECV) measured by Computed Tomography (CT) on the response of primary lesions to preoperative chemotherapy in abdominal neuroblastoma.

**Methods:**

A total of seventy-five patients with abdominal neuroblastoma were retrospectively included in the study. The regions of interest for the primary lesion and aorta were determined on unenhanced and equilibrium phase CT images before treatment, and their average CT values were measured. Based on patient hematocrit and average CT values, the ECV was calculated. The correlation between ECV and the reduction in primary lesion volume was examined. A receiver operating characteristic curve was generated to assess the predictive performance of ECV for a very good partial response of the primary lesion.

**Results:**

There was a negative correlation between primary lesion volume reduction and ECV (*r* = -0.351, p = 0.002), and primary lesions with very good partial response had lower ECV (p < 0.001). The area under the curve for ECV in predicting the very good partial response of primary lesion was 0.742 (p < 0.001), with a 95 % Confidence Interval of 0.628 to 0.836. The optimal cut-off value was 0.28, and the sensitivity and specificity were 62.07 % and 84.78 %, respectively.

**Conclusions:**

The measurement of pre-treatment ECV on CT images demonstrates a significant correlation with the response of the primary lesion to preoperative chemotherapy in abdominal neuroblastoma.

## Highlights

• ECV correlates with the response of the primary lesion to preoperative chemotherapy in abdominal neuroblastoma.
• Lower ECV in neuroblastoma is associated with a greater reduction in primary lesion volume after chemotherapy.
• ECV may serve as a new imaging biomarker for predicting chemotherapy efficacy in neuroblastoma.

## Introduction

Neuroblastoma is a type of cancer prevalent in children and mostly originates from the adrenal medulla and retroperitoneal sympathetic nerve chains. Unfortunately, the delayed onset of symptoms often leads to more cases of neuroblastoma being diagnosed in the late stages. Upfront surgery is hard to carry out on the primary lesion of neuroblastoma in the late stages due to its tendency to encase adjacent vascular structures.^1^^,^^2^ As a component of the preoperative treatment plan, chemotherapy can effectively reduce both the size of the tumor and its overall burden. A previous study demonstrated a correlation between the image-defined risk factors and the response of neuroblastoma to preoperative chemotherapy.^3^ Additionally, the pathological and genetic characteristics of the tumor cells can also impact the effectiveness of preoperative chemotherapy in treating neuroblastoma.^4^, ^5^, ^6^ Aside from tumor cells, the extracellular matrix is also a significant factor in the heterogeneity of neuroblastoma. It provides a microenvironment for tumor cells to grow and interact with each other. Through bidirectional regulation between the tumor cells and the extracellular matrix, the tumor microenvironment is affected, resulting in varying levels of heterogeneity within the tumor.^7^^,^^8^ These findings emphasize the need to investigate the extracellular matrix to gain a deeper understanding of the heterogeneity of neuroblastoma.

Extracellular Volume Fraction (ECV) is a parameter that can be utilized to quantitatively assess the extracellular matrix of tissues by measuring the sum of the extracellular space. This can be achieved by calculating ECV by measuring the Computed Tomography (CT) values of the regions of interest and the blood pool before and after the administration of a contrast agent, along with considering the hematocrit of the patient.^9^, ^10^, ^11^ Fujita et al.^12^ conducted a study that suggested a possible correlation between ECV and the effectiveness of preoperative chemotherapy in patients with pancreatic ductal adenocarcinoma. A previous study demonstrated the significant correlations between CT-based ECV and pathological features in abdominal neuroblastoma.^13^ However, no studies have yet been conducted to investigate the correlation between imaging-based ECV and the response of neuroblastoma to preoperative chemotherapy.

Consequently, the authors conducted a study to explore the correlation between ECV measured by CT and the response of the primary lesion to preoperative chemotherapy in abdominal neuroblastoma.

## Materials and methods

### Patients

**Fig. 1:**
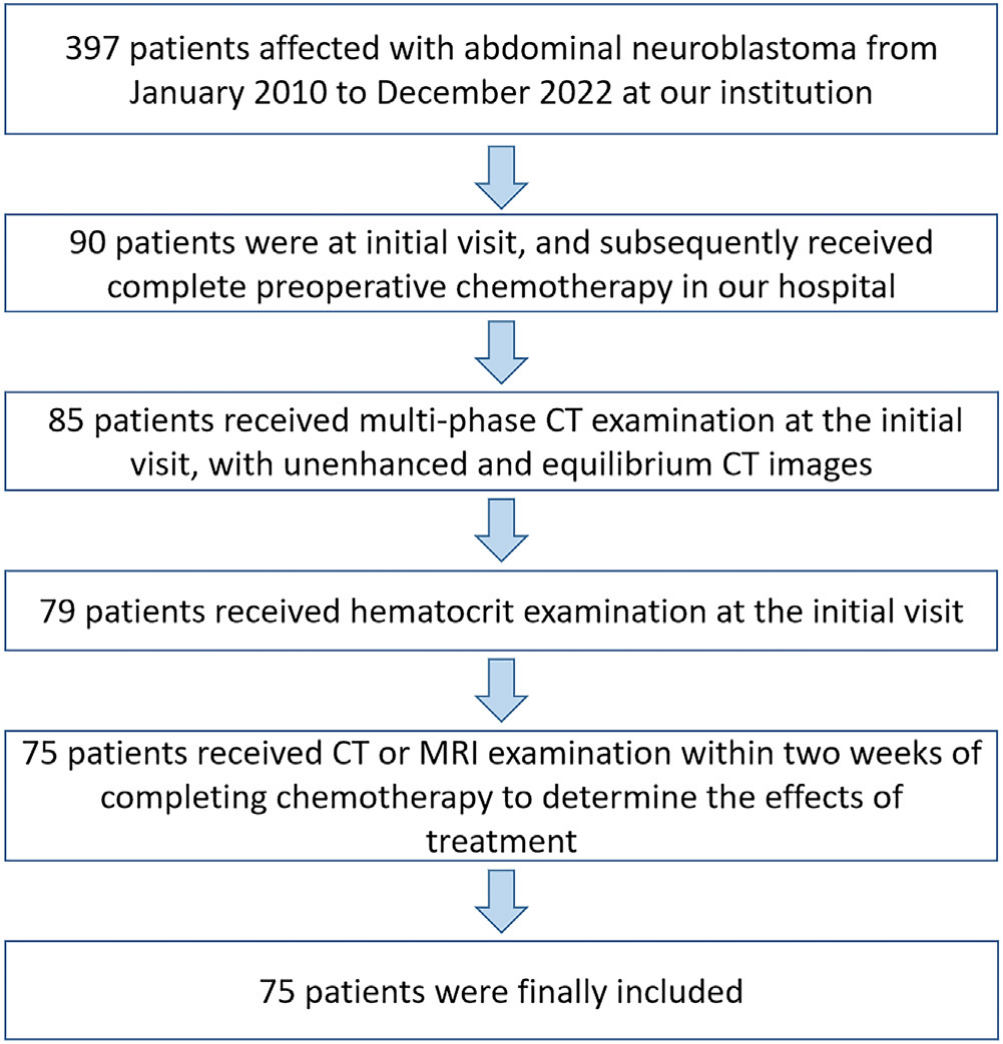
The selection flowchart of patients.

The authors retrospectively collected data from neuroblastoma patients who were admitted to the institution between January 2010 and December 2022, based on predefined inclusion and exclusion criteria. Inclusion criteria for the study were as follows: (1) Patients with abdominal neuroblastoma; (2) Initial visit and subsequent complete preoperative chemotherapy received at the studied hospital; (3) CT examination performed at the initial visit, with unenhanced and equilibrium CT images; (4) Hematocrit index examined at the initial visit; (5) CT or MRI performed within two weeks of completing neoadjuvant chemotherapy to evaluate the treatment response of the primary lesion. Exclusion criteria for the study were as follows: (1) Unclear pathological diagnosis; (2) Prior surgery, radiation therapy, or chemotherapy; (3) Lack of unenhanced and equilibrium CT images at the initial visit; (4) Lack of hematocrit index at the initial visit. A flowchart of patient selection is shown in Fig. 1. This retrospective study was approved by the Institutional Review Board, Children's Hospital of Chongqing Medical University (File nº 202235). This observational study followed the Strengthening the Reporting of Observational Studies in Epidemiology (STROBE) guidelines. Because this study retrospectively collected data from patients who were admitted to the studied institution within the last ten years, there were differences in the drugs used for neoadjuvant chemotherapy among patients. However, in general, neoadjuvant chemotherapy regimens were made according to patients' risk groups using the Chinese Children's Cancer Group Neuroblastoma regimens, with drugs such as Vincristine, Cyclophosphamide, Carboplatin, and Etoposide being primarily utilized.

### CT scanning

The study utilized either the Brilliance iCT (Philips) or Lightspeed VCT (GE Healthcare) scanner. Both unenhanced and equilibrium images were acquired, with the equilibrium images taken 3 minutes after the unenhanced scanning. A nonionic iodine contrast agent was used, with an injection dose of 1.5‒2.0 mL/kg and an injection rate of 0.5‒3.5 mL/s. The tube voltage ranged from 80 to 100 KV, with the tube current automatically adjusted according to the patient's body type. The thickness of the scanning slice was 5.0 mm, and the reconstruction slice was about 1.0 mm.

### Image analysis

**Fig. 2:**
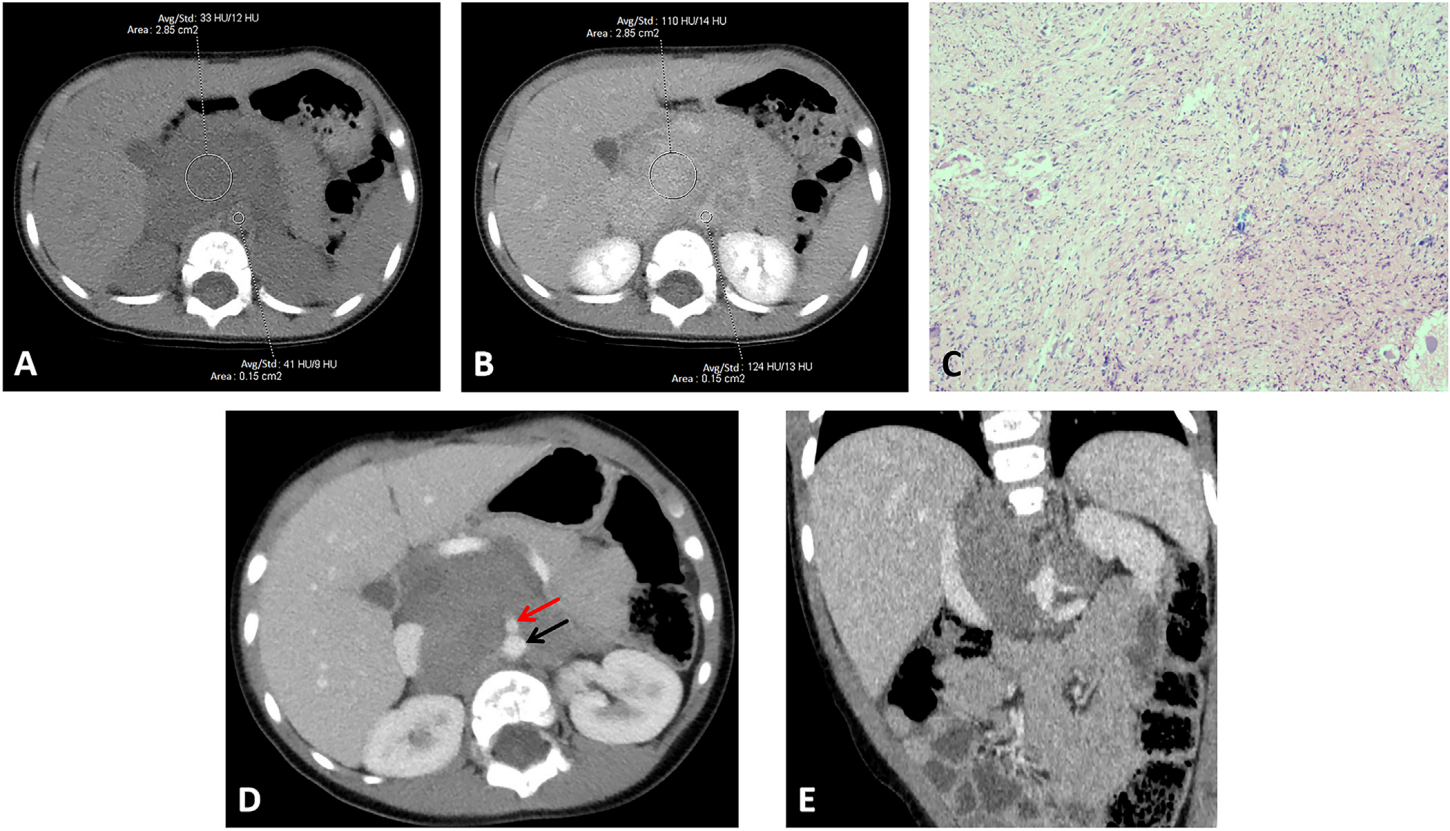
Examples of regions of interest delineation in primary tumor and abdominal aorta at unenhanced phase (A) and equilibrium phase (B) before treatment; hematoxylin-eosin staining (10×10) of the specimen (C). The primary lesion volume reduction of this patient (boy, 1-year-old) was 0.67, which was classified as other response group. The hematocrit of this patient at initial visit was 0.37, so the extracellular volume fraction was 0.58. D and E show the axial and coronal CT images after three course treatment, respectively, and the lesion still encased the superior mesenteric artery (red arrow) and abdominal aorta (black arrow).

Two pediatric radiologists independently conducted image analysis. The primary tumor and abdominal aorta regions of interest were delineated at the same level of the unenhanced and equilibrium CT images before treatment, and their mean CT values were measured. The regions of interest in the primary tumor were delineated at the largest axial slice while avoiding areas with obvious calcification, cystic degeneration, and necrosis. The regions of interest in the abdominal aorta were delineated away from the vessel wall. Fig. 2 illustrates the delineation of the regions of interest in the primary tumor and abdominal aorta. ECV was calculated using the following formula: ECV=(1−hematocrit)×(▵HU_tumor/▵HU_aorta)×100%, where hematocrit was the hematocrit index measured at the initial visit.^10^ Specifically, △HU_tumor was calculated as the CT value of the tumor at the equilibrium stage minus the CT value of the tumor at the unenhanced stage, while △HU_aorta was calculated as the CT value of the abdominal aorta at the equilibrium stage minus the CT value of the abdominal aorta at the unenhanced stage. The volume of the primary lesion before and after chemotherapy was calculated using the elliptic formula, and the reduction in primary lesion volume was determined. Patients were categorized into either the very good partial response group or the other response group based on the reduction in primary lesion volume.^14^ Specifically, a primary lesion volume reduction of between 90 % to 99 % was defined as a very good partial response, while a primary lesion volume reduction below 90 % was defined as the other response.

### Statistical analysis

Statistical analysis was performed using Medcalc software (version 8.0.2). The median (quartile) was used to represent measurement data. Mann-Whitney *U* test was used to compare measurement data between groups, while a chi-square test was used to compare counting data between groups. Spearman correlation analysis was used to determine the correlation between measurement data. The predictive value of ECV for a very good partial response of the primary lesion was evaluated using a Receiver Operating Characteristic (ROC) curve, and the Area Under the Curve (AUC) was calculated along with a 95 % Confidence Interval (95 % CI). The best cut-off value and its corresponding sensitivity and specificity were also calculated. The inter-agreement of ECV measured by two pediatric radiologists was evaluated using the Intraclass Correlation Coefficient (ICC). A Bland-Altman plot was used to visualize the measurements of ECV and primary lesion volume reduction between two radiologists. A p-value of less than 0.05 was considered statistically significant.

## Results

### Patients

This study included a total of 75 patients, with ages ranging from 0.2 to 12 years and a median age of 3.0 (1.0, 4.0) years. Of these patients, 47 were male and 28 were female. Patients received 2 to 11 courses of preoperative chemotherapy, with 4 (37/75, 49.3 %) or 5 (22/75, 29.3 %) courses being the most common. The primary lesion volume reduction after chemotherapy ranged from 0.17 to 0.99, with a median of 0.87 (0.72, 0.95). Among the patients, 29 cases were in the very good partial response group and 46 cases were in the other response group. The clinical features of the included patients are shown in Table 1. The pre-treatment hematocrit ranged from 0.19 to 0.41, with a median of 0.28 (0.26, 0.32), while the pre-treatment ECV values ranged from 0.14 to 0.60, with a median of 0.33 (0.25, 0.41). There was no significant correlation between hematocrit and ECV (*r* = 0.151, p = 0.175). The study found that the ECV for male patients ranged from 0.14 to 0.60, with a median of 0.33 (0.25, 0.43), while in female patients, ECV ranged from 0.22 to 0.51, with a median of 0.33 (0.25, 0.41). There was no significant difference in ECV between males and females (p = 0.913), and no significant correlation was found with age (*r* = -0.056, p = 0.634).

**Table 1:** The clinical features of the included patients.

Clinical features	Results (n = 75)
Age (year)	3.0 (1.0, 4.0)
Gender	
Male	47 (62.67 %)
Female	28 (37.33 %)
Hematocrit	0.28 (0.26, 0.32)
Extracellular volume fraction	0.33 (0.25, 0.41)
Reduction in primary lesion volume	0.87 (0.72, 0.95)
Response of primary lesion	
Very good partial response	29 (38.67 %)
Other partial response	46 (61.33 %)
INRG stage	
L2 stage	14 (18.67 %)
M stage	61 (81.33 %)
MYCN status	
Amplified	19 (25.33 %)
Non-amplified	36 (48.00 %)
Undefined	20 (26.67 %)
Risk group	
Intermediate-risk	14 (18.67 %)
High-risk	61 (81.33 %)

INRG, International Neuroblastoma Risk Group.

### Correlation between primary lesion volume reduction and ECV

**Fig. 3:**
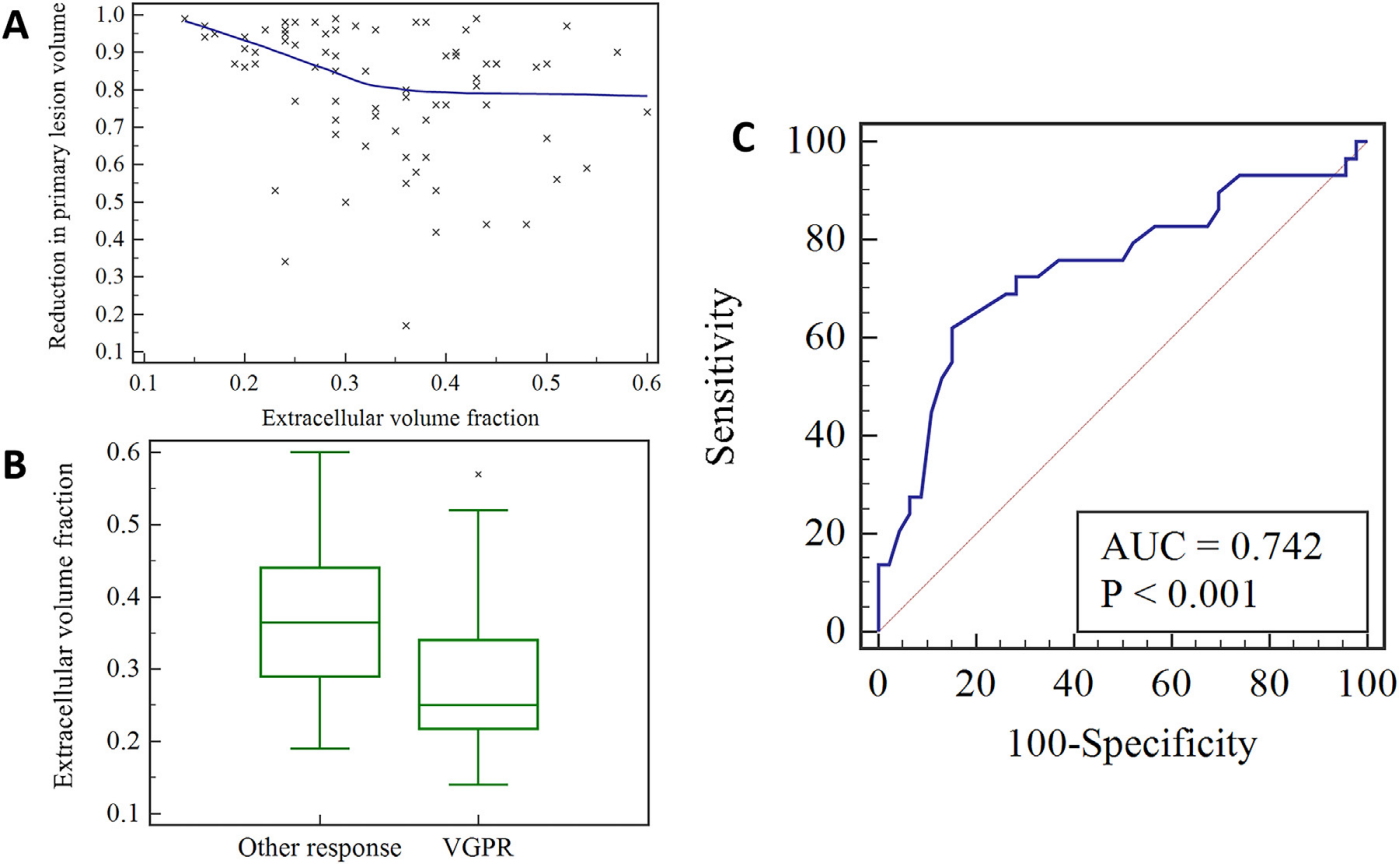
A scatter plot showing the correlation between pre-treatment extracellular volume fraction and reduction in primary lesion volume (A); a box plot comparing the pre-treatment extracellular volume fraction between the very good partial response group and the other partial response group (B); a receiver operating characteristic curve showing the predictive ability of pre-treatment extracellular volume fraction for very good partial response of primary lesion (C). VGPR, Very Good Partial Response.

A significant negative correlation was found between the reduction in primary lesion volume and ECV (*r* = -0.351, p = 0.002), as shown in Fig. 3A. The ECV in the very good partial response group ranged from 0.14 to 0.57, with a median of 0.25 (0.21, 0.35), while that in the other response group ranged from 0.19 to 0.60, with a median of 0.37 (0.29, 0.44). There was a statistically significant difference in ECV between the very good partial response group and the other response group (p < 0.001) (Fig. 3B). The AUC for ECV in predicting the very good partial response of the primary lesion was 0.742 (p < 0.001), with a 95 % CI of 0.628 to 0.836. The optimal cut-off value was 0.28, and the sensitivity and specificity were 62.07 % and 84.78 %, respectively (Fig. 3C).

### Inter-observer evaluation of primary lesion volume reduction and ECV

**Fig. 4:**
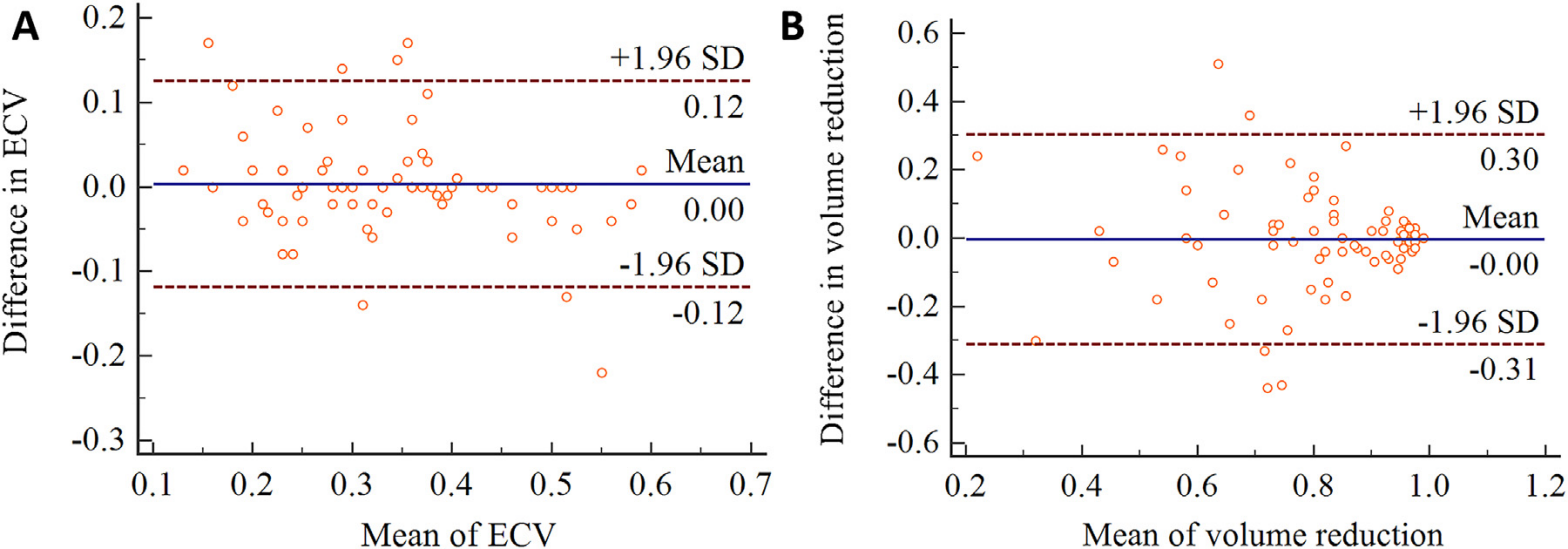
The Bland-Altman plot for extracellular volume fraction (A) and primary lesion volume reduction (B) measured by two radiologists.

The ICC for ECV measured by two pediatric radiologists was 0.854 (95 % CI 0.778‒0.905), while the ICC for primary lesion volume reduction measured by two pediatric radiologists was 0.779 (95 % CI 0.651‒0.861). Fig. 4 shows the Bland-Altman plot for ECV and primary lesion volume reduction measured by two radiologists.

## Discussion

Tumor tissues contain an extracellular matrix that provides a microenvironment that supports and nourishes the growth of tumor cells. In this study, the authors investigated the correlation between ECV measured by CT and the response of primary lesions to preoperative chemotherapy in abdominal neuroblastoma. The results revealed a negative correlation between the reduction in primary lesion volume and pretreatment ECV. Moreover, primary lesions that exhibited a very good partial response had lower ECV levels, indicating that ECV is associated with the response of primary lesions to chemotherapeutic agents in neuroblastoma.

Yanishevski et al.^5^ demonstrated that high-risk neuroblastoma with MYCN gene amplification was more sensitive to preoperative chemotherapy. MYCN gene amplification is associated with increased invasiveness of neuroblastoma and plays a key role in promoting proliferation, invasion, and metastasis of neuroblastoma.^15^^,^^16^ Co-expression of MYCN and Bcl-2 genes has also been shown to induce the secretion and activation of matrix metalloproteinases in neuroblastoma, leading to the degradation of the extracellular matrix.^17^ Neuroblastoma with MYCN amplification has been shown to be more resilient, suggesting a potential effect of MYCN gene amplification on the extracellular matrix of neuroblastoma.^18^^,^^19^ Therefore, it is possible that neuroblastoma with MYCN amplification may contain less extracellular matrix, which could be related to the sensitivity of tumor cells to chemotherapeutic agents. Wang et al.^13^ also found that MYCN-amplified neuroblastoma had a significantly lower ECV. These results indirectly suggest a potential correlation between the extracellular matrix and the response of primary lesions to chemotherapy in neuroblastoma.

In the present study, the authors observed that a lower pre-treatment ECV in neuroblastoma was associated with a greater reduction in primary lesion volume after chemotherapy. Furthermore, the pre-treatment ECV in the very good partial response group was significantly lower than that of the other partial response group, indicating a significant correlation between pre-treatment ECV of neuroblastoma and the response of the primary lesion to preoperative chemotherapy. The extracellular matrix acts as a physical barrier for chemotherapy drugs to enter tumor cells, and a harder and less elastic extracellular matrix can hinder the spread of chemotherapy drugs into tumor cells.^20^^,^^21^ Therefore, neuroblastoma with higher ECV may be less sensitive to preoperative chemotherapy due to the difficulties in drug penetration caused by the extracellular matrix. A previous study indicated that well-differentiated ganglioneuroblastoma contains a higher amount of extracellular matrix,^22^ which may explain why this subtype of neuroblastoma is less sensitive to chemotherapy and exhibits no significant change in tumor volume after chemotherapy. Therefore, measuring ECV before treatment may provide a quantitative evaluation of neuroblastoma sensitivity to chemotherapy drugs. Additionally, these findings suggest that the extracellular matrix may serve as a potential therapeutic target, and remodeling the extracellular matrix could potentially enhance the sensitivity of neuroblastoma to chemotherapy drugs.^23^

This study has several limitations. Firstly, the sample size was small, and the study was conducted at a single center. Therefore, further studies with larger sample sizes and multi-center collaborations are needed to confirm these findings. Secondly, ECV in neuroblastoma was only preliminarily measured on CT images, and it is necessary to further verify the correlation between ECV and neuroblastoma extracellular matrix at the histological level. Future studies should aim to incorporate histological analysis of ECV in neuroblastoma to provide a more comprehensive understanding of the relationship between ECV and the extracellular matrix. Lastly, concerns about radiation dose limit the application of multi-phase CT in children. Zormpas-Petridis et al.^24^ demonstrated the potential of native T1 mapping to assess response to MYCN-targeted treatment in the Th-MYCN mice model of neuroblastoma. Therefore, it is expected that radiation-free MRI techniques, such as T1 mapping, will be used in future studies to further evaluate ECV in neuroblastoma, which will reduce the potential harm associated with radiation exposure.

## Conclusion

In conclusion, the present study suggests that the measurement of ECV on CT images of abdominal neuroblastoma is correlated with the treatment response of the primary lesion to preoperative chemotherapy. This finding indicates that ECV may serve as a new quantitative imaging biomarker for predicting chemotherapy efficacy in neuroblastoma. In the future, more radiation-free imaging parameters associated with the extracellular matrix could be further investigated in pediatric neuroblastoma.

## Declarations

### Ethics approval and consent to participate

This retrospective study was approved by the Institutional Review Board, Children's Hospital of Chongqing Medical University (File nº 202235).

### Consent for publication

Not applicable.

### Availability of data and materials

The datasets generated or analyzed during the study are available from the corresponding author upon reasonable request.

## Authors’ contributions

Haoru Wang, Xin Chen, Ling He: Conceptualization; Methodology. Haoru Wang: Data curation; Writing-Original draft preparation. Haoru Wang, Mingye Xie, Ting Li, Jinjie Qin: Visualization; Investigation. Xin Chen, Ling He: Supervision. Haoru Wang: Validation. Xin Chen, Ling He: Writing-Reviewing and Editing. All authors reviewed the manuscript.

## Funding

The project was funded by the Natural Science Foundation of Chongqing (CSTB 2023NSCQ-BHX0127).

## Conflicts of interest

The authors declare no conflicts of interest.
